# Hôtel Dieu

**Published:** 1831-10

**Authors:** 


### HOSPITAL REPORTS.

### HÔTEL DIEU.

*Fatal Case of Tetanus from a Wound made in the Palm of the Hand by a Splinter of Wood. Tetanic Symptoms proceeding from the Wound towards the Trunk.*

A MAN fell whilst at work upon a log of wood a splinter of which inflicted a slight wound upon the palm of the hand; emollient poultices were applied, and in a short time the wound healed. He was admitted with the following symptoms: The fingers of the right hand firmly closed; muscles of the fore arm affected by involuntary, permanent, and painful contractions, the severity of which was occasionally diminished and increased; no other part of the body was affected; muscles of the jaw not attacked; the cicatrix in the hand was slightly painful; general health good. It was clear that the patient was attacked with tetanus, although the disease commenced in an unusual manner; he was freely bled from the arm, and fifteen grains of watery extract of opium administered, and he was kept in a bath during the greater part of the night.

On the following day, the tetanic contractions were not confined to the upper extremity; the muscles of the posterior part of the trunk participated in the attack. V.S. repeated; warm bath. The cicatrix was attentively examined by M. DUPUYTREN: it was found hard and deep, and M. D. removed it with a bistoury. Below the cicatrix a nervous filament was detected, of which about half an inch was removed: a roll of lint, covered with extract of belladonna, was introduced into, and kept in the wound by bandages.

All these precautions were, however, useless: the muscles of the neck were attacked; deglutition became difficult, but the speech was not impeded, and the levator muscles of the lower jaw, which are usually first attacked, remained unaffected; the intellectual faculties were undisturbed.

The next day, the patient appeared rather better; but, after several violent paroxysms of spasm, he sank rapidly, and died three days after the invasion of the disease.

*Dissection, forty hours after death.* The muscles of the right fore arm were observed to be powerfully contracted, and the fingers were firmly bent inwards. At the bottom of the wound which had been made by the removal of the cicatrix, was seen the divided extremities of the collateral branch of the radial nerve, which passes to the index finger: there was nothing remarkable either in the consistence or colour of this nerve. The median nerve was laid bare throughout its whole extent: it presented in every part a yellowish tinge, which was more evident when it was compared with the radial and ulnar nerves, and with the corresponding nerve of the opposite side.

Head: The dura mater and sinuses were in a state of congestion; the arachnoid and pia mater were slightly red, but this was considered merely as a phenomenon of imbibition.

Spinal column: There was a well-marked ecchymosis on the posterior part of the neck, where the muscles, gorged with black blood, appeared to have been torn: the spinal portion of the dura mater was of a red colour, but this evidently depended upon imbibition. Rather a considerable quantity of sero-sanguineous fluid occupied the arachnoid cavity, and this appearance probably depended upon the position in which the body had been placed, and the time which had elapsed after death: there was nothing peculiar in the consistence or colour of the spinal marrow.

The lungs and heart were in a state of advanced putrescence.

The progress which tetanus followed in this case differs from that which is ordinarily observed: in general, the disease shews itself at a distant point from, and totally independent of the wound; it is commonly by a violent contraction of the levator muscles of the jaw, by trismus, in fact, that the disease commences, and from hence it extends rapidly to other parts of the body. But in this case the disease proceeded from the wound to other parts of the body, which were attacked in succession, leaving quite unaffected that part of the jaw in which it commonly makes its first appearance. Although a very vigorous treatment was promptly adopted, the disease proceeded with its ordinary rapidity: the state of the cicatrix particularly attracted the attention of M. Dupuytren, and it was removed: its hardness and depth led to the suspicion that it was the cause of all the symptoms, by keeping up irritation upon some nervous filament. It might also have happened that some particles of wood remaining in the wound, over which cicatrization had taken place, gave rise to the tetanic symptoms. Cases are recorded by different authors, in which foreign bodies, thus situated in the tissue, have produced the disease, which has ceased upon their removal. In a case of tetanus treated by M. Dupuytren, in which the disease arose from a wound on the fore arm, inflicted by a violent blow of a whip, a part of the lash was found, upon examination after death, at the bottom of the wound. This foreign body lay upon the radial nerve, and must therefore have caused great and dangerous irritation during life; and to this circumstance was attributed the formidable symptoms under which the patient had laboured.

The lesions observed after death in the above case throw no light on the nature of the disease. The ecchymosed and contused state of the muscles on the back of the neck may be considered as indicative of the violent contractions which had existed there, and which frequently distend the muscular fibres to such a degree as to rupture them in different parts. The fact which most claims attention is the yellow colour of the median nerve, which extended to the upper part of the arm: no such appearance was seen in the radial or cubital nerve of the same side, nor in the median nerve of the other extremity: but although this fact is well worth noticing, no conclusion can be formed from it, without justifiably incurring the charge of precipitation, which should be so carefully avoided upon such subjects. It may be well to state that this yellow tinge of the nerves is frequently found in those which, during life, have been affected with inflammation, and that it has been often seen in patients who have died during the existence of very painful neuralgia of the sciatic nerve: but, on the other hand, it should be remembered that one of the branches of the median nerve had been removed to a certain extent, and perhaps this circumstance had some connexion with the unusual colour of the principal nervous trunk.

